# Understanding the human-cattle relationship in mountain cow-calf systems: Insights from Spanish farmers

**DOI:** 10.1017/awf.2025.10012

**Published:** 2025-06-23

**Authors:** Laura X Estévez-Moreno, Gustavo A María, Pilar Santolaria, Morris Villarroel, Genaro C Miranda-de la Lama

**Affiliations:** 1 Department of Agricultural Sciences and the Environment, University of Zaragoza, Zaragoza, Spain; 2 Department of Animal Production & Food Science, University of Zaragoza, Zaragoza, Spain; 3 CEIGRAM, ETSIAAB, Technical University of Madrid (UPM), Madrid, Spain

**Keywords:** Animal welfare, cattle behaviour, cow-calf systems, farmers’ perceptions, handling, human-animal relationship

## Abstract

In livestock production, animal welfare is shaped by the quality of the relationship between animals and the farmers with whom they interact. This study investigated the perceptions of Spanish farmers of extensive mountain cow-calf systems regarding the impact of human intervention on animal behaviour, with attention to the human-animal relationship (HAR), farm management strategies, and interactions between animals and unfamiliar people. The research was conducted within the context of cow-calf systems managed under extensive mountain conditions. Seven focus group discussions (FGDs) were conducted, involving 60 mountain cattle farmers from Aragon, Navarra, and the Basque Country. The findings of our study demonstrate that the quality of HAR is contingent upon a multitude of variables, which exert a profound influence on the duration, intimacy, and strength of these relationships, particularly in response to environmental conditions, as reflected, for example, in the changing frequency of human-animal interactions during periods of outdoor grazing compared to times of confinement. Farmers also showed interest in understanding animal behavioural responses in different management scenarios to adapt and individualise their strategies. Across all FGDs, participants recognised the farmer’s role in influencing animals to be less reactive or fearful toward humans through their handling style. Finally, the interactions between people and cows extend beyond farmers and their families, incorporating a variety of unfamiliar individuals who also play a role in each cow’s relationships with humans. Therefore, farmers’ perceptions reveal that animals’ responses to humans are not homogeneous but are highly dependent upon context, ontogeny and levels of familiarity.

## Introduction

Animals under human care are exposed to a potentially large range of both familiar and unfamiliar humans (Williams *et al.* 2024). The human-animal relationship (HAR) is dynamic and multifactorial, defined as the reciprocal perception of animals and humans reflected in their mutual behaviour and feedback in every interaction (Waiblinger *et al.* 2006). Recognition of the role of the HAR in cattle welfare has led to the development of a robust and growing body of evidence from all over the world, regarding the consequences of this relationship, primarily for animals (Raussi 2003). Multiple disciplinary and methodological approaches have shown that attitudes and perceptions of stock people towards cattle have effects on the level of stress and fear animals experience, as well as their behavioural responses, productivity, health, and farm profitability (Hemsworth 2003; Ceballos *et al.* 2018; O’Leary *et al.* 2018; D’Aniello et al. 2022; Adler *et al.* 2019). There is a growing interest in studying farmers’ perceptions as regards several issues related to farm animal welfare (Balzani & Hanlon 2020). Nevertheless, these studies have concentrated more on the cow-human relationship in the context of dairy production systems (e.g. Bertenshaw & Rowlinson 2009; Maher *et al.* 2021) than on beef-oriented ones (Dwane *et al.* 2013). Moreover, the behavioural responses of cows to human handling are typically investigated through experimental or on-farm studies, employing standardised testing procedures (Shahin 2018; Wellbrock & Knierim 2020).

Cow-calf extensive or semi-extensive farming systems represent an essential component of beef production in Spain, with a census of more than two million cows (Ministerio de Agriculura, Pesca o Alimentación - MAPA 2024). Although these systems are replicated throughout the Spanish geography, the production of calves in these breeding systems has a special relevance in mountain systems, such as *Picos de Europa*, the Central System, the Pyrenees and the Iberian System (Barrantes *et al.* 2009). These systems are based on extensive grazing in mountain regions and on native breeds that are relatively hardy, adapted to local climatic and geographical conditions (Collantes 2003; Rodríguez Bermúdez et al. 2019). During the grazing season, farms located in these areas combine the use of valley meadows near villages, mid-mountain pastures and high mountain pastures (passes), so the feeding of sucker cows is almost exclusively based on fresh forage or silage from the region (Ministerio de Agriculura, Pesca o Alimentación - MAPA 2024). Normally, cows feed on mid-mountain pastures during the spring, and as summer sets in, they move upwards until they reach the pass. In the fall, they are moved back down to the mid-mountain areas, where they return to graze on the regrowth of what they consumed in the spring. Once winter arrives, the animals are either stabled in the winter barn or the herd is relocated to an alternative area with more clement conditions and/or greater access to pasture (Muñoz-Ulecia *et al.* 2021). This practice, which involves transporting cattle for several days on foot, is known as transhumance and has been legally regulated in Spain since 1273 (Baena & Casas 2010). The aforementioned conditions influence the frequency and nature of farmer-cow interactions, which may fluctuate significantly throughout the year. These interactions are more intense when the animals are kept indoors or near the barn, in contrast to what occurs during the grazing season. Nevertheless, by retaining the suckler cows for several years, farmers can observe their animals’ behavioural responses to both their own handling and interactions with other people all year long and throughout the animals’ lives, under these changing management conditions.

In recent years, livestock farming systems and farmers’ working conditions have undergone significant changes due to economic, health, environmental, ethical, and reputational challenges (Beaujouan *et al.* 2021). A major issue has been a crisis of credibility of livestock farmers among segments of developed societies (Henchion *et al.* 2022), driven by the disconnection between citizens and rural life, the livestock sector’s lack of transparency as regards animal welfare, and the stigmatisation of farmers’ economically driven relationships with animals (Duley *et al.* 2022). Nevertheless, several studies have shown that HARs extend beyond the economic interests of farmers, also involving cultural and emotional dimensions (Ibañez & Mol 2024; Garber & Turner 2025). These aspects are shaped by sociodemographic factors, the number and species of animals managed, the chosen farm management strategy, and the farm’s productive orientation (Crawshaw & Piazza 2022; Johanssen *et al.* 2023). Together, these elements influence the frequency, type, and duration of human-animal contact. Despite growing interest in farmer-animal relationships, there is still limited research on how these relationships unfold in mountain livestock systems, where distinct environmental, social, and productive conditions may shape unique interaction patterns. By exploring farmer-cow relationships in this context, the present study seeks to address this gap and provide insights that can inform strategies to enhance both human and animal welfare. Therefore, this study aims to investigate the perceptions of Spanish farmers engaged in extensive mountain cow-calf systems regarding the impact of human interactions and management on cow behaviour, with particular attention to three key areas: (i) farmer-cattle relationships; (ii) farm management strategies; and (iii) interactions between animals and unfamiliar people.

## Materials and methods

### Ethical status

The objectives of the study were submitted to the Research Ethics Committee of the Autonomous Community of Aragon (CEICA), which recommended that the study be conducted in accordance with the guidelines of the Declaration of Helsinki. In line with these ethical guidelines, particular attention was given to the application of principles related to informed consent, ensuring that participants received clear and understandable information regarding the study prior to taking part; respect for participants’ autonomy in deciding whether to participate and remain in the study; fairness and equity in the selection process; and the protection of participants’ privacy and well-being.

### Positionality statement

This study focuses on the farmers’ perspective on human-animal relationships, making it necessary for us to explicitly state our experiences and potential biases, as these may influence the interpretation of the data. Acknowledging these perspectives ensures greater transparency and rigour in our conclusions. Authors of this study are men and women holding doctoral degrees in agricultural science and animal production sciences. We are researchers and university professors affiliated with the University of Zaragoza and the Technical University of Madrid. Our shared research focuses on farm animal welfare, encompassing species such as fish, goats, sheep, cattle, pigs, rabbits, and poultry, as well as the perceptions and attitudes of various social stakeholders as regards this issue. Our work combines research and teaching, which involves the observation and direct handling of different farm animal species, and interacting with farmers; however, none of us has professional experience as livestock farmers. The welfare of farm animals is closely connected to that of the humans involved in their rearing, transportation, marketing, pre-slaughter handling, and slaughter. Through this study, we aim to provide an integrative perspective on the diversity and complexity of human-cow interactions within cow-calf systems and, more broadly, in other livestock systems operating in rural areas. By adopting the perspective of farmers, themselves, we hope to offer new insights into the human factors — including, but not limited to, farmers — that influence the outcomes of human-animal interactions regarding animal behaviour.

### Participants and procedure

This study was carried out with farmers from the mountainous regions of the Autonomous Community of Aragon, Basque Country and Navarra, in northern Spain. It used focus group discussions (FGD), to collect data concerning participants’ knowledge, perceptions and opinions as regards the role of humans in the behavioural responses of cows during the human-animal interactions, using an inductive and exploratory approach. The FGD is a qualitative research method where a small group of participants discuss a particular issue under the guidance of a moderator who keeps the discussion focused, non-threatening and as ‘natural-feeling’ as possible, with minimal self-involvement (Wibeck *et al.* 2007). As such, this method: (i) facilitates the gathering of information that arises from participants’ interaction; (ii) helps them to recall experiences and ideas that would not arise without such interaction; and (iii) enhances the identification and understanding of shared perceptions and disagreements (Williamson 2018). The sample size was determined by combining pragmatic and interpretative criteria, following the recommendations of Braun and Clarke (2021) and the concept of information power (Malterud *et al.* 2016). Given the exploratory nature of the study, the aim was to capture a wide diversity of perspectives and experiences among the participating cattle farmers, while also ensuring the presence of certain shared features within each group to support mutual understanding and fluid discussion. To this end, FGDs were conducted in several regions and participants were recruited according to the following inclusion criteria: (i) aged at least 18 years; (ii) currently working as a cattle owner; (iii) with a minimum of three years breeding experience; and (iv) being directly involved in the handling of animals. Participants were recruited through the local farmers’ associations, with an initial invitation informing them of the following: (i) they would join a group session with other farmers to talk about how they handle their cows and how the animals respond to human interaction; (ii) the session would last between 40 min and 1.5 h; and (iii) their participation would be voluntary and not linked to any incentives. To ensure fairness and equity, participants were selected solely according to the above criteria, without discrimination based on gender, age, or other social characteristics. The final sample, consisting of 60 farmers (58 men and two women with ages ranging from 22 to 68 years) distributed across seven FGDs (Table 1), provided sufficient and in-depth information to address our research objectives. The number of participants per FGD ranged from seven to eleven people, which is considered an adequate group size to allow for participation and discussion (Krueger & Casey 2014). The most common breeds described by the FGD participants were the native *Pirenaica* and *Parda de Montaña* (Brown Alpine or *Braunvieh*) breeds. However, there are other breeds, including the Simmental (*Fleckvieh*), Charolais and Limousin, which were also represented.

**Table 1.** Location and main breeds of cattle reared by Spanish farmers (n = 60) participating in the focus group discussions (FGD) of the study.

Code	Location	Participants	Main breed reared
FGD1.	Aragon (Province of Huesca- Ainsa)	8	*Pirenaica*
FGD2.	Navarra	9	*Pirenaica*
FGD3.	Basque Country (Province of Alava)	11	*Pirenaica*
FGD4.	Aragon (Province of Teruel)	9	*Pirenaica*
FGD5.	Aragon (Province of Huesca – Broto Valley)	8	*Parda de Montaña*
FGD6.	Aragon (Province of Huesca – Broto Valley)	7	*Parda de Montaña*
FGD7.	Aragon (Province of Huesca – Broto Valley)	8	*Parda de Montaña*

### Data collection

At the beginning of each FGD, the moderator (LXE) and the assistant (GCM) (both researchers of the study) introduced themselves and informed: (i) the aim of the study and of the FGD; (ii) the expected duration of the session; (iii) that participation was voluntary and not linked to any incentives; (iv) that participants were free to leave the session at any time without needing to provide an explanation; (v) that participants were free to express their opinions at any time during the FGD, but were not required to answer every question posed by the moderator; vi) that the audio of the entire session would be recorded; (vii) that all data from the focus groups would be processed anonymously, and any information that could identify a specific participant or third party would be removed from the transcripts prior to analysis; and (viii) that the data collected would be used exclusively for research purposes. Informed verbal consent was obtained from the participants in each session. LXE facilitated the discussion reading out trigger questions included in the discussion guide (Table 2) and encouraging participation of all group members. Furthermore, there were specific questions asked by LXE when it was deemed necessary to maintain certain dynamics, explore divergent perspectives, or delve into ideas that arose. The assistant (GML) monitored the total duration of each FGD and took notes on the main topics and ideas discussed. Each FGD lasted between 50 and 70 min.

**Table 2.** Semi-structured discussion guide used to facilitate the six focus group discussions on farmers’ perceptions of the role of human intervention in animal behaviour.

Stage	Content
Introductions (5–10 min)	LXE, GML Participants: Livestock Breed: Herd Size (optional) Knowledge Acquisition (how farmers learned about farming and livestock management) General management strategy throughout the year (location and grazing conditions)
Personal experience: farmer-cattle relationships and behavioural responses (20–30 min)	What strategies do you use to make your cows react with docility to human handling? How do you think the way you relate to your cows affects the way they react to humans? What other factors besides the farmer’s handling affect the cows’ behaviour towards humans? What determines whether cows react one way or another to [the factor mentioned]?
Other human-animal interactions (20–25 min)	How do your cows behave to handling by people other than the farmer, such as a veterinarian? Which other people interact with the cows besides you? In what situations or contexts do cows usually interact with [each type of person mentioned]? How do cows usually react to proximity or contact with [each type of person mentioned]? What might cause cows to react in this way?
Closing (5 min)	Summary of the main points discussed Final comments Acknowledgments to the farmers for them for their involvement

### Data analysis

The *verbatim* transcriptions of all FGD (listed as 1 to 7) were made, with a code assigned to each participant (P1, P2, P3…), to ensure anonymity. All personal data and identifying references were removed from the transcripts. A qualitative thematic analysis of transcriptions was performed following the methodology proposed by Braun and Clarke (2006) and Braun *et al.* (2019). Both the transcriptions and qualitative thematic analysis were conducted in Spanish, the native language of all focus group participants, as well as the moderator (LXE) and the assistant (GCM) who carried out the data analysis. Some interviewee quotes were translated and included here to help illustrate the study’s findings. A native English-speaking researcher, proficient in both English and Spanish, translated the quotes to ensure they accurately conveyed the meaning, context, and intent of the speakers.

The thematic analysis was conducted using an inductive and reflexive approach (Braun *et al.* 2019) to deepen the understanding of farmer-cattle relationships and their effects on animal behaviour from the farmers’ perspective, recognising that these relationships can develop over many years, vary by context, and evolve over time. According to the inductive approach, the process of coding and defining themes and subthemes was carried out iteratively and collaboratively between two researchers of the study. In this sense, the themes were not predetermined by a prior theoretical structure, but on the contrary, the entire analysis was guided by the data. Consequently, the thematic analysis performed comprised several stages based on the methodology outlined by Braun and Clarke (2006) and Braun *et al.* (2019).

The researchers individually read the transcripts from all focus groups and took initial notes on potential codes (words or short phrases). They then jointly established the basic guidelines for code format and wording, and prepared a preliminary codebook that evolved throughout the coding process. To define the strategy for making modifications to the codebook, the two researchers collaboratively coded segments from different transcriptions before beginning the individual coding process, which was conducted simultaneously and in the same order. The coding of each focus group was discussed, and the codebook, themes and sub-themes defined through an iterative process. Following the individual coding of each focus group, the researchers jointly reviewed the changes that each one made to the codebook. They first identified codes that matched either fully or partially, unifying the wording after confirming alignment in their subjacent meanings and descriptions. Next, they examined excerpts related to non-matching codes to understand the basis of the differences, ultimately reaching a coding consensus. Then, based on these discussions, each researcher revisited the coding performed, made necessary revisions, thereby developing independently a thematic structure of themes and sub-themes, clustering the codes into shared meaning-based patterns. Both researchers shared the proposed thematic structure and discussed it to agree on an updated version that would serve as the basis for the next round of coding. Once all the focus groups were coded, the thematic structure was consolidated collaboratively between the two researchers, and relevant quotes were identified to illustrate the themes and subthemes.

## Results

**Figure 1.**
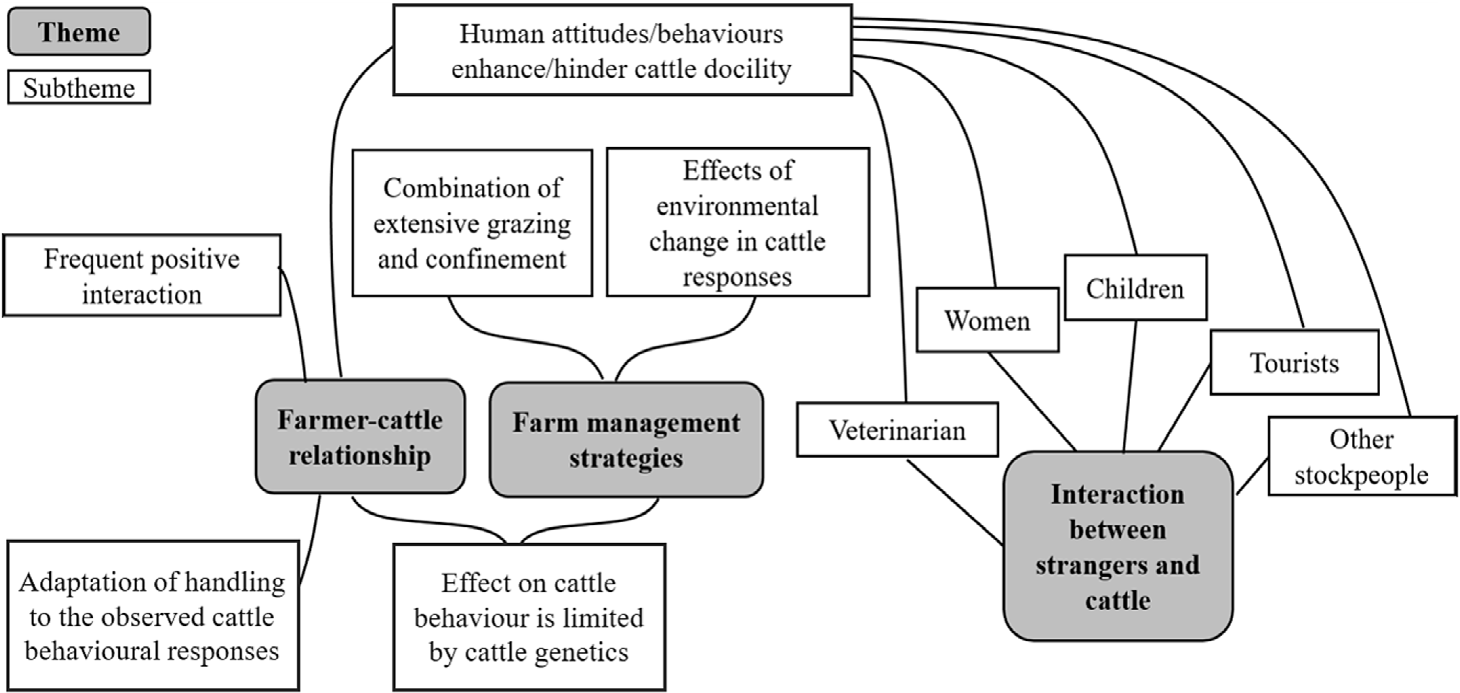
Thematic map of the themes and subthemes identified from six focus groups involving a total of 60 farmers, investigating their perceptions regarding the role of human intervention in animal behaviour. The white boxes represent subthemes organised within the three main themes outlined in the grey boxes: farmer-cattle relationship; farm management strategies; and interactions between strangers and cattle. The full lines indicate the connections between themes and subthemes, while the dashed lines represent the relationships among the subthemes.

The perceptions of Spanish farmers engaged in extensive mountain cattle systems regarding the impact of human interactions and management on livestock behaviour were elucidated through FGDs in three central themes: (i) farmer-cattle relationship; (ii) farm management strategies; and (iii) interactions between unfamiliar people and cattle (Figure 1).

### The farmer-cattle relationship

The leading role of farmers’ handling style in realising a scenario whereby animals were less reactive or fearful toward humans was recognised in all the FGDs. Although farmers acknowledged the existence of a multitude of potential scenarios, as “*each farm is a world in itself*” (FGD6; P6), they delineated numerous attitudes that inform their relationship with livestock and shape the “*what should be*” farmer behaviour towards animals. All participants were in unanimous agreement that farmers should act in a calm, patient and knowledgeable manner, avoiding any actions that might cause distress to the cattle, such as shouting, physical violence or sudden movements. In all the focus groups, farmers frequently mentioned that gentle handling, which includes no mistreatment, enhances the animals’ docile temperament. Hence, the positive behaviour of the farmers towards cattle was stated as a factor underlying the calm handling that promotes cows’ tameness, while shouting or hitting animals was considered counterproductive, since it stimulates fear behaviours and, in situations of high stress, can trigger aggressive behaviours. Hence, one important strategy mentioned was to “*handle cows with love*” (FGD2; P1) and behave calmly while handling them:

*“It is also true that the personality of the farmer is very important, there are farms that we know very well, where the cattle are very calm, farmers do not use a stick… I mean that these cattle, if you treat them with affection and you have that communication with cows, that is, like with people, and you give them that affection, they also give you back that docility, that easy handling…”* [FGD3; P3].

Although the description ‘easy to handle’ was the most common way for farmers to characterise a docile animal, some also described it as a quiet or calm animal, as opposed to one that was very nervous or reactive. Additionally, several farmers emphasised that treating animals with kindness and care was not typically difficult to achieve, since most of them love all their cattle (not just their favourites), some even as if the cows were family members. In most FGDs participants pointed out that a farmer must constantly observe the behaviour of cows, both when they are isolated and when they are in the herd, to be able to choose or adapt handling to improve their cows’ behavioural responses. This means making animals non-reactive and non-aggressive when handled and making handling easier and safer for both humans and animals.

Some participants believed that their proficiency in raising cattle occurred as a direct result of their passion for animal husbandry. Others, however, attributed their expertise to the experience they have accumulated over time. As a farmer from the FGD2 explained:

“*it has been hard for me to understand, but I believe that a farmer is the psychologist of his/her animals. You are always observing them, and they somehow wait for your reaction*” [P6].

Farmers from all FGDs agreed that frequent contact with cows enables them to become familiar with the farmer’s presence, making it easier to carry out all the daily tasks (e.g. moving the herd from one grazing place to another, cleaning the stable, filling the feeders, etc). In addition, in FGD5 and FGD6, participants emphasised that this contact must be associated with positive stimuli (e.g. stroking, scratching or feeding), which may help to strengthen the bond, even in management strategies where the frequency of contact is very low. In FGD6 farmers also stressed that frequent contact with animals during their first year is an especially useful strategy for building a strong positive farmer-animal relationship. Furthermore, one farmer mentioned that the first week of a calf’s life and weaning are also special occasions to strengthen such bonds.

In FGD4, an additional discussion took place regarding whether farmers should spend more time with the most nervous and reactive animals, which are considered problematic due to the greater challenges they pose during routine handling and the increased risk of accidents. However, no consensus was reached. Some emphasised that petting, handling, and talking to these animals, mainly while they were in the winter stable, would help make them more docile and easier to handle over time. Nevertheless, other farmers felt that a strategy of deliberately apportioning more handling time to specific animals was ineffective and they did not consider their animals’ temperament during handling. In the FGD1 and FGD5 groups, they identified handler personality as representing a major factor in determining the quality of the HAR during routine handling. It was observed that cows on a farm with multiple handlers exhibited behavioural differences during handling, contingent on the handlers’ approach. It is postulated that this phenomenon is attributable to the inherent personality traits and the quality of time devoted to the animals by specific handlers. One illustrative example mentioned during one of the focus groups is as follows:

*“My son, who has been working with me for a year, spends a lot of time with the heifers, which I have never done. I have a very strong personality, I mean, the animals also notice that. Now he tells me that I constantly shout and that he is calmer, and the truth is that he does better with young animals, but he spends a lot of time with them…”* [FGD5; P4].

### Farm management strategies

In general, farmers in all FDGs acknowledged that the time they spend with their cows depends on farm management strategies. However, they also noted that these strategies are contingent upon a multitude of factors, including the season, herd size, the availability of public or private grazing areas, their location and accessibility, and farmer preferences. For example, some farmers maintain their cattle in meadows and pastures where snow is absent throughout the year. Others may pasture their cattle in areas in close proximity to the barn, while still others relocate their animals to mountain valleys situated several hours drive from the barn. A number of participants indicated that the frequency of visits to their herds during the grazing season varied according to the herd size, distance and ease of access to the grazing areas. It was reported that this frequency can range from daily or several times a week, to a few times throughout the entire season. Furthermore, the frequency of visits during the grazing season also varies over time, depending upon the distance between the grazing locations and the herds. On average, farmers participating in this study owned approximately 60 cattle, with a maximum of 120 cows. Although most farmers agreed that the mentioned conditions determine the possibilities of maintaining a close relationship with animals, some others mentioned that cows’ behaviour is mainly ‘defined at home, in the stable’. Indeed, some have suggested that the duration of cows’ confinement in stalls or paddocks adjacent to farms has significant consequences for farmers seeking to enhance their comprehension of the animals’ responses to handling and to improve HARs. The majority of farmers were in accordance that when animals are left for extended periods without human contact, they are more likely to exhibit heightened reactivity compared to those handled on a daily basis. This phenomenon is particularly evident in animals that are born free-ranging and spend their initial months in grazing areas (FGD1, FGD5). Moreover, some farmers in FGD4 also indicated that they could attempt to treat their reactive cows more closely and empathetically, but elect not to do so, as they perceive doing so would put them at risk of injury.

In some groups, the movement of cattle through grazing areas was brought up as a process where contrasting cattle behaviours can be observed. First, there was consensus that animal movements within the farm, either between grazing areas or in the barn, do not involve special handling difficulties, but it is always evident which animals are the most fearful. In terms of grazing in open areas, comments offered two alternate stances. First, there was a consensus that, under critical environmental conditions, the movement of animals can be carried out easily. For example, when feed is scarce and the weather becomes harsher, the animals react quickly by following the farmer, moving in the direction indicated by them or getting on the truck. Meanwhile, alternatively, a number of participants highlighted various scenarios with the potential to elicit differential behavioural responses in animals that complicate herd movement: (i) when the farmers’ helpers have changed; (ii) when a cow hides the calf in the brush; (iii) when two or more herds meet on the road; (iv) when unexpected encounters between cows and unfamiliar individuals, such as tourists, occur while the cows are grazing; (v) when the docks in the mountain ports are in bad condition; (v) when there is still an abundant supply of food, as “*if you want to move them but the grass is tall, they won’t pay much attention to you*” (FGD1; P4). For example, a participant in FGD5 elucidated that there may be some old cows that, when they feel comfortable grazing “*become strong, run away and do not want to come back*” (FGD 5; P3), and in the midst of their reaction affect the behaviour to other younger ones, hindering the collection process.

Farmers in FGD1, FGD3, and FGD4 also discussed the actual scope of farm management strategies to generate changes in animal behaviour throughout ontogeny. Farmers agree that cows have a personality that remains consistent throughout their lives, which means that the same type of handling applied to the herd will elicit different responses depending on the individual differences among the animals. Furthermore, while farmers acknowledged that certain behaviours can be moderated through management, it was their contention that if an animal is reactive or fearful it will exhibit more pronounced reactions throughout its life when faced with unusual stimuli or situations, compared with a docile animal. For example, in FGD4, farmers mentioned:

1. *“Well, I guess everyone handles them differently, but if you put them in small places when they are small, or whatever, and you handle them, carry them, touch them, stroke them, pamper them, they will love you more.”*
2. *“Yes, but if you do that to ten animals, there will be one or two that will not respond.”*
3. “*And besides, if you leave them in the wild, forgotten during months…”*
4. “*Well, but that happens to all of us…”*
5. “*That’s not only with cows. If we are confined for ten hours… when we get out some of us will be better than others”.*
6. Various: “*Yes, yes…”*

### Interactions between unfamiliar people and cattle

When asking about the extent to which humans can affect the behavioural reactions of cows, all FGDs concluded that cattle tend to exhibit different behaviours when in the presence of unfamiliar people. Although participants were asked specifically about veterinarians, other types of people were mentioned, such as various members of the farmer’s family (especially women and children), transporters or traders, and other people.

#### (1) Veterinarians

These professionals were considered able to trigger the most reactive responses in cattle, regardless of breed. In all the FGDs, farmers accepted and justified that fear of veterinarians, as they are generally associated with painful handling. *“The day the veterinarian comes and they are tied for example in the stock, all cows get nervous, they already know that something is going to happen…”* (FGD3; P7). There was also a consensus that one should expect different behaviour towards a farmer or a veterinarian, even if both are carrying out the same procedure, simply because the latter are unfamiliar. However, farmers pointed out that the cows’ reaction to veterinarians also depends on the specific characteristics of the latter, e.g. their skills, their confidence and their calmness when handling the animals. In their opinion, the veterinarian’s behaviour towards animals can lead to situations of relative calm or great stress for animals as well as for farmers and veterinarians. An example of such a stressful situation is described below by a farmer from FGD 2:

*“What I value most in a cow is that if a person approaches the barn with fear, the cow notices. And this year I had a gentleman* [veterinarian] *that came to me and as soon as I entered the barn he said, ‘bring me a package of straw’. I thought: ‘he’s scared’. Then he said to me - ‘tie the cows’, and I said to him - ‘hey, hey, hey, take it easy, eh, these cows are going to retaliate’. He took the tail and the first time, to stick it in, he stuck the needle in six times… and when I told him: ‘The next cow you catch by the tail, if you don’t prick her four times, you’ll leave my barn’. And he said, ‘Are you threatening me?’ – ‘No, the cow is threatening you because she saw that with the fear you have, this is not your profession.’”*

At the other extreme, a farmer who is also a veterinarian pointed out that the results of any handling in terms of the cows’ behavioural response will depend as much on the veterinarian as on the farmer. He explained that if the farmer is nervous when the veterinarian is in the barn, the whole situation will become more stressful, so it may be preferable for the farmer to leave the barn for the duration of the procedure.

#### (2) Other family members

When referring to other family members who occasionally visit the barn, participants in all FGDs mentioned women and children. There was a consensus amongst all FGDs that there might be differences in the behaviour of the animals towards the usual handlers, generally men, and the women in the family. However, they also agreed that these differences are associated more with the fact that they are non-regular visitors to the farm and not specifically with their gender. In fact, in FGD5 and FGD6, it was emphasised that gender aside, the calmer style of women may facilitate cow handling compared to men, as long as women do not show fear. The idea being that fear in humans is perceived by animals and can trigger reactions of reduced docility. Another farmer clarified that his wife, who usually handles the animals, has a “*different style, but they* [cows] *treat us the same*”, while another farmer who handles cows alone pointed out that “*if my wife or my daughter or whatever comes in, be careful that the doors are not closed because*…” (FGD7; P7). In this final remark, the farmer highlighted the potential risks for his wife, daughter, or others who are not usually around the cows, associated with entering the barn. He emphasised the importance of ensuring that the pen doors remain open so that these individuals can leave safely if needed. This reflects a concern specific to this particular farmer regarding unfamiliar people and does not represent a consensus from the farmers in general.

The presence of children in the barn was perceived as a potentially risky situation by the majority of farmers, given that cows often exhibit reactive behaviours, such as fleeing, or even aggressive responses, like lunging in response to visual and/or auditory contact with children. Furthermore, several farmers indicated that these behaviours may manifest even when the children in question are members of the farmer’s own family. Certain participants provided even more elaborate interpretations of these behaviours. These included the suggestion that cows may perceive children as similar to dogs (or small predators), that they are repelled by the high-pitched tone of children’s voices, their rapid movements, their gaze, or that the animals feel they can dominate them. Some farmers also discussed whether the reaction of cows was due to the particular characteristics of the children, to the fact that they were unfamiliar people, or to a combination of both. Another discussion concerned the children’s age. Some farmers reported that young children (up to 4 years old in FGD5 and up to 5 or 6 years old in FGD7) provoke defensive or aggressive reactions from the cows but as the children gain height compared to the animals, cows’ behaviour may change “*because the cow stops seeing the child as small*” (FGD7; P7). In this regard, some participants pointed out that during their own childhood this situation was not so marked, given that the handling of animals used to be more frequent, as well as their contact with cows. In this regard, one of the older farmers explained: “*When I was a child I almost lived under or over a cow… but now my children have too many activities and only come to the stable very rarely*” (FGD3; P4).

#### (3) Occasional handlers

In addition to the veterinarian, occasional handlers mentioned by farmers included hauliers, operators involved in pre-slaughter logistics in the slaughterhouse, and personnel responsible for the care and management of livestock at cattle exhibitions. The effects of the handling of these individuals on cattle behaviour were not discussed in depth in the FGDs, except in the case of workers involved in loading and unloading animals from trucks: some farmers mentioned that these people usually have a lot of experience, as they are used to working with all types of animals, which would facilitate any kind of handling. Other farmers elucidated that the dearth of knowledge regarding the animals to be handled by occasional handlers renders processes such as loading or unloading more intricate. “*I tell him* [the driver], *‘stay there and I’ll load all the cows on the truck’. It is much easier to do it alone than with other people*” (FGD2; P5). Furthermore, participants in FGD6 and FGD7 commented about the convenience of handling the cows themselves to avoid unnecessary stress and fear-related behavioural responses caused by other people during loading and unloading. All agreed that regardless of the temperament of the animals, the conditions of the infrastructure (handling chutes, loading ramps, trucks) can have an important impact on the behavioural reactions of cows.

#### (4) Visitors and tourists

Although there was not much mention of people other than those already described, this group could include visitors or tourists who come across the herds grazing in the mountains, or people from the towns through which the herds pass. The relationship between these people and the cows was described by farmers in three different contexts. Firstly, when the animals are grazing freely, passing hikers or tourists usually do not present a problem, even when in the midst of several cows. However, the risk of a fear reaction or attack increases if people try to interact with cows, touch them, or take pictures too close to them, especially if the cows have calves. Second, the apathy of cows towards tourists can potentially give rise to fear-related behaviours when tourists are accompanied by dogs (particularly if the dogs are not tethered) or when they cycle in close proximity to the animals. And, thirdly, when animals are in the barn, the arrival of tourists often triggers fear reactions. According to one farmer, this is because they are stabled and have fewer opportunities to escape from a possible risky situation. Also, when cows are in places where painful handling occurs (e.g. physical restraint for veterinary treatments), they react more strongly to unfamiliar people. In order to avoid meeting unfamiliar people, participants of FGD 6 and FGD7 explained that it moving animals through different grazing zones in the early morning or later at night could be useful.

## Discussion

Extensive cattle farming, and cow-calf systems in particular, play a pivotal role in the environmental, economic and social sustainability of the mountainous regions of Spain (Ruiz *et al.* 2020). This is due to the significant contribution they make to the retention of the rural population, the preservation of local culture, the assurance of food security, the maintenance of biodiversity, and the conservation of landscapes (Muñoz-Ulecia *et al.* 2024). In this context, our study is one of the first to contribute to the understanding of the complexity and scope of HAR in the context of Spanish mountain livestock farming. According to the farmers’ perspective, these relationships encompass not only farmers and their families, but also key people such as veterinarians, occasional handlers, as well as visitors and tourists. Furthermore, different styles of herd handling and farm management strategies can significantly influence the nature of HARs.

### The farmer-cattle relationships

Overall, participants’ perceptions of the human-cattle relationship and its effects on cattle behaviour are largely consistent with the extensive body of scientific evidence on the topic. All participants concurred that the calm and gentle handling of cattle encourages docile behaviour, as previously observed by Destrez *et al.* (2018). Additionally, farmers emphasised the significance of knowledge and skills pertaining to cattle handling and management, as well as an empathetic disposition towards cows, as factors that foster favourable behavioural responses in livestock and mitigate the risk of accidents during handling (Adler *et al.* 2019). The results of our study indicate that farmers are aware of the significance of understanding cow behaviour in both group and individual contexts during specific phases of handling. This finding is noteworthy because it demonstrates that farmers possess the capacity to interpret behavioural variations across different handling scenarios. Moreover, the participants acknowledged that regular, positive, and intimate contact with calves during their initial months of life is crucial for fostering a close HAR, as evidenced by the experimental findings of Wada *et al.* (2021). Farmers are keenly aware of the dynamic nature of the relationships they have with their animals and employ a continuous feedback process to foster these relationships. This process enables farmers to modify their handling techniques in accordance with the animal’s behavioural responses, thereby reinforcing the bond between the two. It seems reasonable to posit that the farmers’ capacity and inclination to utilise diverse techniques when interacting with their animals is enhanced by experience, which in turn affects the outcome of the HAR.

### Farm management strategies

The productive cycles that are characteristic of cow-calf systems afford farmers the opportunity to observe, over time, the effects of different management practices on both specific individuals and the herd as a whole. This, in turn, allows for the development of a more refined HAR that is aligned with the specific characteristics and needs of each individual animal, thereby facilitating more effective and adaptive management strategies. This contrasts with feedlot systems, where weaned calves, yearling heifers or bulls typically remain on the farm for less than a year. Furthermore, the relatively small scale of beef cattle farms in Spain which, according to MAPA (2024), rarely exceed 100 head (as in the case of our study), allows farmers to implement more detailed and personalised management strategies for each animal. These findings are in accordance with those of Buddle *et al.* (2021) and Ventura *et al.* (2023), who propose that HAR in cattle ranching may be influenced by farm size. Furthermore, these authors posit that farmers on smaller farms may cultivate more intimate and familiar relationships with their cattle in comparison to those on larger farms. From the results presented, it can be inferred that the close contact that farmers maintain with their cows during their first year of life, coupled with their capacity to modify management practices to enhance behavioural responses or foster relationships with specific animals (e.g. preferred cows), indicates that farmers are capable of discerning their cows’ individual characteristics and developing profound emotional attachments towards them. This may constitute a strategic approach to improve herd management, as it allows for the timely recognition and attention to specific behavioural problems that may affect the health and welfare of particular animals in a herd. Furthermore, our results indicate the existence of an enduring emotional bond between farmers and their animals, which manifests in two distinct dimensions over time. The first emotional bond is formed with a specific individual, typically a favourite cow. The second is established with the entire herd, regardless of which individuals are added or removed (e.g. sold, discarded, died). In terms of the initial dimension, the results indicate that farmers can cultivate deep and lasting emotional connections with selected suckler cows, potentially reaching a level of intimacy comparable to that observed between humans and their companion animals (Wilkie 2005). This is especially pertinent considering that cows often remain on these farms for a period of more than ten years. Regarding the second dimension, the results illustrate how farmers can reconcile their feelings with the eventual departure of the animal.

According to farmers, cow-calf production systems in the mountain environment are heterogeneous, being conditioned by climatic and geographic conditions, as well as the differential access of farmers to different resources. Our results also suggest that these systems create particular conditions for the HAR and for cows’ welfare since they combine at least two very contrasting handling conditions: (i) extensive grazing in open fields, where cows have freedom for movement and social interaction, and the possibility to express behaviours such as grazing (Alsaaod *et al.* 2022); and (ii) confinement during the winter months, when animals are comfortable regarding weather and access to feed but are physically more restricted and experience the most stressful situations related to competition and social conflict (Miranda-de la Lama *et al.* 2013a; Smid *et al.* 2020). Under the former conditions, the farmer’s contact with animals tends to be infrequent but linked to positive or neutral stimuli (talking, observing, giving food), whereas with the latter, the farmer may have daily, close and positive contact with animals, but also performs the most stressful handling (Williams *et al.* 2024). This contrasting scenario may be advantageous for animal welfare when compared to fully extensive systems, where cattle are less likely to habituate to handling or develop a positive or neutral HAR (Creamer & Horback 2021), or cow-calf intensive systems, with animals constantly kept in confinement (Bertelsen & Vaarst 2023). Therefore, under seasonal housing conditions (e.g. winter), farmers can be more certain about which animals are docile or reactive to handling during sensitive periods such as calving or weaning (Estévez-Moreno et al. 2021b). However, further research is required to determine the impact of different levels of farmer-cow interaction throughout the year on animal behaviour, compared to stall or extensive rearing.

Another interesting result that arose from the FGDs is that farmers recognise the importance of the environment as a mediating factor in the HAR. In particular, through observing and handling animals during grazing, they may establish links between resource availability and animal behaviour. Such behaviour can, in turn, serve as an indicator of pasture conditions (Fernández-Giménez & Fillat 2012). In light of the phenomenon of climate change, this is a crucial consideration, as it affects the annual availability of pasture and water, influencing grazing patterns and frequently compelling farmers to alter their grazing areas (Cassidy 2012; Fernández-Giménez & Fillat 2012) as well as imposing additional animal handling and management challenges regarding the annual distribution of grazing and confinement, the management of animals in mountainous areas, or the operational and logistical decisions involved in the movement of herds (both in trucks and on foot). The recognition by farmers of the importance of genetics in animal temperament is an aspect that has been previously documented in this type of system (Estévez-Moreno *et al.* 2021b), and it has been shown that genetic selection or choice of breed stock within a farm is almost certainly an important aspect to improve livestock responses to humans (Beaujouan *et al.* 2021).

### Interactions between cattle and unfamiliar people

One major consensus of farmers is that animals show a differentiated behavioural response to familiar as opposed to unfamiliar people. In particular, according to the farmers in our study, unfamiliar people can elicit behavioural reactivity, although this is influenced by the setting of the interaction and the personality traits of the unfamiliar person (e.g. nervous, reactive, noisy, curious, patient, etc). While these results have been documented in the extant literature on livestock HAR with known humans (e.g. Ellingsen *et al.* 2014), they have not been studied with unfamiliar humans and livestock, although they have been inferred between humans and zoo animals (Patel *et al.* 2019). In this context, the results obtained here demonstrate a broader and more complex scenario regarding the construction of HARs in extensive mountain systems (compared to intensive systems), by identifying possible categories of people interacting with livestock. The farmers grouped these unfamiliar people by combining common characteristics such as gender, age and the type of interaction they have with the animals.

According to farmers, veterinarians are perceived as unfamiliar people by cows, even though they may have occasional contact. The role of the veterinarian on these farms is traditionally limited to handling that requires isolation, restraint, and painful procedures that may be stressful. Nevertheless, the threshold of their behavioural responses in these situations may be associated with conditioned ramp aversion (Creamer & Horback 2021), or with the cognitive ability of certain cows to remember certain people or events (Jardat & Lansade 2022). Moreover, it is not uncommon for other individuals to be present during veterinary treatments, including farmers, workers, attendants, and onlookers. These individuals may contribute to the animal’s stress levels through physical restraint, visual, tactile, and olfactory contact. In general, there is a paucity of research examining veterinarians’ perceptions of the behavioural responses of animals during clinical management and their perceptions of the effect of the farmer’s husbandry on cow behaviour (Norring *et al.* 2014). Participants also noted that handling by unfamiliar personnel during transport operations can make herding, handling, and loading/unloading more difficult. They linked these situations to behaviours related to fear and stress. This aligns with evidence suggesting that handling and loading can be more stressful than other stages of the transport process, such as the journey itself, often leading to anxiety, frustration, and fear (see EFSA Panel on Animal Health and Welfare et al. 2022; Grandin 2024). This is particularly relevant given that, although these handling practices are transient in relation to the animal’s lifespan, they combine novel circumstances and disparate, unpredictable livestock handling techniques that can be markedly stressful for the animals, affecting their welfare and even the safety of the individuals involved (Losada-Espinosa *et al.* 2020).

Farmers identified women and children as family members who do not have regular contact with the animals and can be unfamiliar people to the cows. This phenomenon may be attributed to the gender division of labour, which may result in women assuming responsibility for the rearing of animals other than cattle, such as laying hens, goats or sheep (Hovorka 2012), or working in various off-farm jobs (Offenhenden & Soronellas-Masdeu 2021; Castelló & Romano 2023). The gender composition of the FGDs partly reflects the reality of livestock production in the region. According to the latest data from the Spanish Agricultural Census (Instituto Nacional de Estadistica - INE 2020), men are the majority of holders of farms specialising in cattle rearing and/or fattening, as well as those specialising in the production of both milk and beef. However, although men often remain the main decision-makers on livestock farms, Spanish women are playing an increasing role in the livestock sector (Fernández-Giménez *et al.* 2021). Interestingly, the farmers in our study acknowledged the existence of different levels of women’s involvement in livestock farming and commented on the experience of women developing their own handling style (associated with greater peace of mind) by developing a close relationship with the cows. Furthermore, the greater sensitivity and empathy of Spanish women (meat consumers, meat retailers) towards farm animal welfare compared to men has been verified in several studies (Miranda-de la Lama *et al.* 2013b; Estévez-Moreno *et al.* 2021a), but behaviours and attitudes in the context of livestock production, especially beef production, have not yet been studied. A limitation of the present study is that it was not possible to differentiate between men’s and women’s perceptions of their relationship with cows and their possible effect on cow behaviour. However, our findings reinforce the need for further research into the human-cattle relationship and its impact on both human and animal welfare from a gender perspective.

Concerning children, our study suggests various aspects that could determine their relationship with cattle, such as their behaviour, age, and height, as well as the similarity between children and potential carnivores (e.g. dogs) and the effect of habituation of animals to the constant contact with children. For pet dogs, younger children have been reported as being less able to interpret certain animal behaviours which will have implications for the animal’s quality of life (i.e. anxiety, frustration and rough contact; Lakestani *et al.* 2014; Hall *et al.* 2019). In the case of children living on dairy cattle farms in Wisconsin (USA), Lee *et al.* (1997) found that the attitudes of parents who allowed children to be near a cow’s hindquarters were strongly influenced by their desire to help their children gain respect for animals and understand animal behaviour. Conducting further studies on children’s relationships with cows, particularly in livestock households, would provide evidence to create safer, enriching environments that promote positive interactions for both children and animals. This should be approached from the perspectives of applied ethology and anthrozoology.

The growth in tourism in Spain’s mountainous regions over the past three decades has prompted a shift in livestock farming practices to more remote grazing areas, accompanied by a decline in the number of available workers willing to engage in livestock-related activities. However, this has also created opportunities for additional income generation among livestock farming households (O’Rourke *et al.* 2016). Such income may be derived from a variety of sources, including farm tours, rural tourism rental homes (Muñoz-Ulecia *et al.* 2021), or off-farm family work. In this scenario, the opportunity for encounters between cows and tourists is increasing. According to our results, it is very likely that these encounters do not represent risks for either the animals or the tourists, and measures are implemented to minimise such risks (e.g. moving the animals during the night). Nevertheless, it is conceivable that the conjunction of tourists’ favourable or unfavourable perceptions of cattle and cows, their motives for visiting mountainous regions, their degree of awareness regarding cattle management, and the potential for conflict with the presence of urban dogs (e.g. unleashed), may engender a spectrum of tourist behaviours towards cows that could elicit negative behavioural responses from the animals. Despite the paucity of research examining the consequences of these relationships for both animals and humans, this topic merits particular scrutiny due to its potential to shape public opinion and, in turn, precipitate a decline in tourism. This, in turn, could have a knock-on effect on small-scale rural economies, including those reliant on services such as catering, accommodation, and hiking.

Finally, our results highlight the need to analyse in more detail, the interactions of farm animals with non-familiar people, considering factors such as the context within which these interactions occur, the type of persons involved, the type of interaction, and the objective of the interaction. Additionally, it is necessary to investigate the association of the aforementioned factors with people’s attitudes toward animals, because our findings indicate that these attitudes may have a greater influence on the outcome of interactions than gender, age, or appearance. This deeper analysis of non-familiar humans – cows would not only provide valuable insights into human-farm animal interactions but also help identify and propose practices that foster safer interactions for both animals and humans.

## Animal welfare implications and Conclusion

This study sheds light on the complex and multidimensional nature of the HAR in extensive mountain cow-calf systems, emphasising the critical role it plays in shaping herd welfare. The ability of farmers to interpret and respond to individual and herd behaviours in these management conditions reflects a deep understanding of animal welfare and management. This skill is rooted in experiential, emotional, and knowledge-based components, all of which are strengthened over time as relationships with animals evolve throughout the farming profession. These factors should be taken into account when developing future strategies or recommendations to enhance HARs involving farmers. Our findings highlight that management techniques and farmers’ attitudes play a crucial role in fostering docility and reducing fear, underscoring the importance of calm and empathetic interactions. They also emphasise the influence of seasonal dynamics on HAR, with confinement facilitating closer interactions and grazing seasons allowing for the expression of natural behaviours. Moreover, the study underscores the significance of observing animals’ behavioural responses during each interaction and adapting management styles accordingly as a vital strategy to foster positive HARs. Interactions between cattle and unfamiliar people, which could affect animal welfare by triggering additional fear and stress in the animals and causing accidents that impact both parties, also underscores the necessity for adaptive management strategies. Farmers’ perspectives on cattle behavioural responses to interactions with veterinarians highlight the importance of calm handling and veterinary skills in reducing animal stress and minimising risks. These insights also stress the crucial role of effective communication between farmers and veterinarians to ensure the welfare of both animals and humans during these interactions.

## References

[r1] Adler F, Christley R and Campe A 2019 Invited review: Examining farmers’ personalities and attitudes as possible risk factors for dairy cattle health, welfare, productivity, and farm management: A systematic scoping review. Journal of Dairy Science 102(5): 3805–3824. 10.3168/jds.2018-15037 30852027

[r2] Alsaaod M, Dürr S, Iten D, Buescher W and Steiner A 2022 Locomotion behavior of dairy cows on traditional summer mountain farms in comparison with modern cubicle housing without access to pasture. PLoS One 17(3): e0264320. 10.1371/journal.pone.0264320 35263371 PMC8906619

[r3] Baena PM and Casas R 2010 Past, present and future of transhumancia in Spain: Nomadism in a developed country. Pastoralism 1(1): 73–90. 10.3362/2041-7136.2010.005

[r4] Balzani A and Hanlon A 2020 Factors that influence farmers’ views on farm animal welfare: A semi-systematic review and thematic analysis. Animals 10(9): 1524. 10.3390/ani10091524 32872206 PMC7552314

[r5] Barrantes O, Ferrer C, Reiné R and Broca A 2009 Categorization of grazing systems to aid the development of land use policy in Aragon, Spain. Grass and Forage Science 64(1): 26–41. 10.1111/j.1365-2494.2008.00666.x

[r6] Beaujouan J, Cromer D and Boivin X 2021 From human–animal relation practice research to the development of the livestock farmer’s activity: an ergonomics–applied ethology interaction. Animal 15(12): 100395. 10.1016/j.animal.2021.100395 34844187

[r7] Bertelsen M and Vaarst M 2023 Shaping cow-calf contact systems: Farmers’ motivations and considerations behind a range of different cow-calf contact systems. Journal of Dairy Science 106(11): 7769–7785. 10.3168/jds.2022-23148 37641296

[r8] Bertenshaw C and Rowlinson P 2009 Exploring stock managers’ perceptions of the human—animal relationship on dairy farms and an association with milk production. Anthrozoös 22(1): 59–69. 10.2752/175303708X390473

[r9] Braun V and Clarke V 2006 Using thematic analysis in psychology. Qualitative Research in Psychology 3: 77–101.

[r10] Braun V and Clarke V 2021 To saturate or not to saturate? Questioning data saturation as a useful concept for thematic analysis and sample-size rationales. Qualitative Research in Sport, Exercise and Health 13(2): 201–216. 10.1080/2159676X.2019.1704846

[r11] Braun V, Clarke V, Hayfield N and Terry G 2019 Thematic analysis. Handbook of Research Methods in Health Social Sciences pp 843–860. 10.1007/978-981-10-5251-4_103

[r12] Buddle EA, Bray HJ and Ankeny RA 2021 Of course we care! A qualitative exploration of Australian livestock producers’ understandings of farm animal welfare issues. Journal of Rural Studies 83: 50–59. 10.1016/j.jrurstud.2021.02.024

[r13] Cassidy R 2012 Lives with others: Climate change and human-animal relations. Annual Review of Anthropology 41: 21–36. 10.1146/annurev-anthro-092611-145706

[r14] Castelló E and Romano MJ 2023 Shepherdesses: new representations of rural women in Spain. Feminist Media Studies 23(4): 1659–1675. 10.1080/14680777.2021.1992646

[r15] Ceballos MC, Sant’Anna AC, Góis KCR, Ferraudo AS, Negrao JA and da Costa MJP 2018 Investigating the relationship between human-animal interactions, reactivity, stress response and reproductive performance in Nellore heifers. Livestock Science 217: 65–75. 10.1016/j.livsci.2018.08.001

[r16] Collantes F 2003 La ganadería de montaña en España, 1865-2000: Historia de una ventaja comparativa anulada. Historia Agraria: Revista de Agricultura e Historia Rural 31: 141–167. [Title translation: Mountain livestock farming in Spain, 1865-2000: The story of a comparative advantage nullified].

[r17] Crawshaw C and Piazza J 2022 How conflicted are farmers about meat? Livestock farmers’ attachment to their animals and attitudes about meat. Psychology of Human-Animal Intergroup Relations 1: 1–19. 10.5964/phair.8513

[r18] Creamer M and Horback K 2021 Researching human-cattle interaction on rangelands: challenges and potential solutions. Animals 11(3): 725. 10.3390/ani11030725 33799955 PMC8000822

[r19] D’Aniello B, Mastellone V, Pinelli C, Scandurra A, Musco N, Tudisco R and Lombardi P 2022 Serum oxytocin in cows is positively correlated with caregiver interactions in the impossible task paradigm. Animals 12(3): 276. 10.3390/ani12030276 35158600 PMC8833709

[r20] Destrez A, Haslin E and Boivin X 2018 What stockperson behavior during weighing reveals about the relationship between humans and suckling beef cattle: A preliminary study. Applied Animal Behaviour Science 209: 8–13. 10.1016/j.applanim.2018.10.001

[r21] Dwane AM, More SJ, Blake M, McKenzie K and Hanlon AJ 2013 Farmers’ self-reported perceptions and behavioural impacts of a welfare scheme for suckler beef cattle in Ireland. Irish Veterinary Journal 66(1): 1–11. 10.1186/2046-0481-66-1 23339820 PMC3599887

[r22] Duley A, Connor M and Vigors B 2022 Irish cattle farmers’ experiences and perceptions of negative framing of farm animal welfare in the media. Irish Journal of Agricultural and Food Research 61(2): 332–346. 10.15212/ijafr-2022-0009

[r23] EFSA Panel on Animal Health and Welfare (AHAW), Nielsen SS, Alvarez J, Bicout DJ, Calistri P, Canali E, Drewe JA, Garin-Bastuji B, Rojas JLG, Schmidt CG, Michel V, Chueca MAM, Padalino B, Pasquali P, Roberts HC, Spoolder H, Stahl K, Velarde A, Viltrop A, Winckler C, Earley B, Edwards S, Faucitano L, Marti S, Miranda de la Lama GC, Nanni Costa L, Thomsen PT, Ashe S, Mur L, Van der Stede Y and Herskin M 2022 Welfare of cattle during transport. EFSA Journal 20(9): e07442. 10.2903/j.efsa.2022.7442 36092766 PMC9449995

[r24] Ellingsen K, Coleman GJ, Lund V and Mejdell CM 2014 Using qualitative behaviour assessment to explore the link between stockperson behaviour and dairy calf behaviour. Applied Animal Behaviour Science 153: 10–17. 10.1016/j.applanim.2014.01.011

[r25] Estévez-Moreno LX, María GA, Sepúlveda WS, Villarroel M and Miranda-de la Lama GC 2021a Attitudes of meat consumers in Mexico and Spain about farm animal welfare: A cross-cultural study. Meat Science 173: 108377. 10.1016/j.meatsci.2020.108377 33308897

[r26] Estévez-Moreno LX, Miranda-de la Lama GC, Villarroel M, García L, Abecia JA, Santolaria, P and María GA 2021b Revisiting cattle temperament in beef cow-calf systems: Insights from farmers’ perceptions about an autochthonous breed. Animals 11(1): 82. 10.3390/ani11010082 33466326 PMC7824783

[r27] Fernández-Giménez ME and Fillat F 2012 Pyrenean pastoralists observations of environmental change: an exploratory study in Los Valles Occidentales of Aragón, Pirineos. Revista de Ecología de Montaña 167: 143–163. 10.3989/Pirineos.2012.167007

[r28] Fernández-Giménez ME, Oteros-Rozas E and Ravera F 2021 Spanish women pastoralists’ pathways into livestock management: Motivations, challenges and learning. Journal of Rural Studies 87: 1–11. 10.1016/j.jrurstud.2021.08.019

[r29] Garber P and Turner S 2025 Illustrating farmer–animal entanglements and emotions: Drawing elicitation in upland Vietnam. Tijdschrift Voor Economische en Sociale Geografie 116(1): 41–54. 10.1111/tesg.12642

[r30] Grandin T 2024 Behavioural principles of handling beef cattle and the design of corrals, lairages, races and loading ramps. Livestock Handling and Transport pp 92–121. CABI: Wallingford, UK. 10.1079/9781800625136.0005

[r31] Hall SS, Finka L and Mills DS 2019 A systematic scoping review: What is the risk from child-dog interactions to dog’s quality of life? Journal of Veterinary Behavior 33: 16–26. 10.1016/j.jveb.2019.05.001

[r32] Hemsworth PH 2003 Human–animal interactions in livestock production. Applied Animal Behaviour Science 81(3): 185–198. 10.1016/S0168-1591(02)00280-0

[r33] Henchion MM, De Backer CJ, Hudders L and O’Reilly S 2022 Ethical and sustainable aspects of meat production; consumer perceptions and system credibility. New Aspects of Meat Quality pp 829–851. Woodhead Publishing: London, UK.

[r34] Hovorka AJ 2012 Women/chickens vs. men/cattle: Insights on gender–species intersectionality. Geoforum 43(4): 875–884. 10.1016/j.geoforum.2012.02.005

[r35] Ibáñez R and Mol A 2024 Joaquín les gusta: on gut-level love for a lamb of the house. Ethnos 89(4): 723–740. 10.1080/00141844.2022.2052926

[r36] Instituto Nacional de Estadística–INE 2020 *Censo Agrario 2020.* https://www.ine.es/dyngs/INEbase/en/operacion.htm?c=Estadistica_C&cid=1254736176851&menu=ultiDatos&idp=1254735727106 (accessed 8 May 2025).

[r37] Jardat P and Lansade L 2022 Cognition and the human–animal relationship: a review of the sociocognitive skills of domestic mammals toward humans. Animal Cognition 25(2): 369–384. 10.1007/s10071-021-01557-6 34476652

[r38] Johanssen JRE, Kvam GT, Logstein B and Vaarst M 2023 Interrelationships between cows, calves, and humans in cow-calf contact systems—An interview study among Norwegian dairy farmers. Journal of Dairy Science 106(9): 6325–6341. 10.3168/jds.2022-22999 37419741

[r39] Krueger RA and Casey MA 2014 Focus Groups: A Practical Guide for Applied Research*, Fifth Edition.* SAGE Publications: New York, NY, USA.

[r40] Lakestani NN, Donaldson ML and Waran N 2014 Interpretation of dog behavior by children and young adults. Anthrozoös 27(1): 65–80. 10.2752/175303714X13837396326413

[r41] Lee BC, Jenkins LS and Westaby JD 1997 Factors influencing exposure of children to major hazards on family farms. The Journal of Rural Health 13(3): 206–215. 10.1111/j.1748-0361.1997.tb00844.x 10174611

[r42] Losada-Espinosa N, Estévez-Moreno LX and Miranda-de la Lama GC 2020 Stockpeople and animal welfare: compatibilities, contradictions, and unresolved ethical dilemmas. Journal of Agricultural and Environmental Ethics 33(1): 71–92. 10.1007/s10806-019-09813-z

[r43] Maher JW, Clarke A, Byrne AW, Doyle R, Blake M and Barrett D 2021 Exploring the opinions of Irish dairy farmers regarding male dairy calves. Frontiers in Veterinary Science 8: 635565. 10.3389/fvets.2021.635565 33959649 PMC8093389

[r44] Malterud K, Siersma VD and Guassora AD 2016 Sample size in qualitative interview studies. Qualitative Health Research 26(13): 1753–1760. 10.1177/1049732315617444 26613970

[r45] Ministerio de Agricultura, Pesca o Alimentación – MAPA 2024 Estudio del sector vacuno de carne español. Análisis de datos SITRAN. Segmento Vacas Nodrizas. Subdirección General de Producciones Ganaderas y Cinegéticas, Dirección General de Producciones y Mercados Agrarios: Madrid, Spain. [Title translation: Study of the Spanish beef cattle sector: Analysis of SITRAN data. Suckler cow segment]

[r46] Miranda-de la Lama GC, Pascual-Alonso M, Guerrero A, Alberti P, Alierta S, Sans P, Gajan JP, Villarroel M, Dalmau A, Velarde A, Campo MM, Galindo F, Santolaria MP, Sañudo C and María GA 2013a Influence of social dominance on production, welfare and the quality of meat from beef bulls. Meat Science 94(4): 432–437. 10.1016/j.meatsci.2013.03.026 23618738

[r47] Miranda-de la Lama GC, Sepúlveda WS, Villarroel M and María GA 2013b Attitudes of meat retailers to animal welfare in Spain. Meat Science 95(3): 569–575. 10.1016/j.meatsci.2013.05.046 23797014

[r48] Muñoz-Ulecia E, Bernués A, Casasús I, Olaizola AM, Lobón, S and Martín-Collado D 2021 Drivers of change in mountain agriculture: A thirty-year analysis of trajectories of evolution of cattle farming systems in the Spanish Pyrenees. Agricultural Systems 186: 102983. 10.1016/j.agsy.2020.102983

[r49] Muñoz-Ulecia E, Martín-Collado D, Bernués A, Peral AT, Casasús I and Villalba D 2024 Can traditional management practices help mountain livestock farms in the Spanish Pyrenees cope with climate change? Regional Environmental Change 24(1): 15. 10.1007/s10113-023-02170-8

[r50] Norring M, Wikman I, Hokkanen AH, Kujala MV and Hänninen L 2014 Empathic veterinarians score cattle pain higher. The Veterinary Journal 200(1): 186–190. 10.1016/j.tvjl.2014.02.005 24685101

[r51] Offenhenden M and Soronellas-Masdeu M 2021 Mountain tourism and agriculture at the crossroads: the case of Cerdanya and Val d’Aran (Catalan Pyrenees). International Journal of Tourism Anthropology 8(4): 304–319. 10.1504/IJTA.2021.123195

[r52] O’Leary NW, Bennett, RM, Tranter RB and Jones PJ 2018 The extent that certain dairy farmer attitudes and behaviors are associated with farm business profitability. Journal of Dairy Science 101(12): 11275–11284. 10.3168/jds.2017-14307 30268625

[r53] O’Rourke E, Charbonneau M and Poinsot Y 2016 High nature value mountain farming systems in Europe: Case studies from the Atlantic Pyrenees, France and the Kerry Uplands, Ireland. *Journal of Rural Studies* 46: 47–59. 10.1016/j.jrurstud.2016.05.010

[r54] Patel F, Whitehouse-Tedd K and Ward SJ 2019 Redefining human-animal relationships: An evaluation of methods to allow their empirical measurement in zoos. Animal Welfare 28(3): 247–259. 10.7120/109627286.28.3.247

[r55] Raussi S 2003 Human–cattle interactions in group housing. Applied Animal Behaviour Science 80(3): 245–262. 10.1016/S0168-1591(02)00213-7

[r56] Rodríguez-Bermúdez R, Fouz R, Miranda M, Orjales I, Minervino AHH and López-Alonso M 2019 Organic or conventional dairy farming in northern Spain: Impacts on cow reproductive performance. Reproduction in Domestic Animals 54(6): 902–911. 10.1111/rda.13446 31006148

[r57] Ruiz FA, Vázquez M, Camuñez JA, Castel JM and Mena Y 2020 Characterization and challenges of livestock farming in Mediterranean protected mountain areas (Sierra Nevada, Spain). Spanish Journal of Agricultural Research 18(1): e0601. 10.5424/sjar/2020181-14288

[r58] Shahin M 2018 The effects of positive human contact by tactile stimulation on dairy cows with different personalities. Applied Animal Behaviour Science 204: 23–28. 10.1016/j.applanim.2018.04.004

[r59] Smid AMC, Weary DM and Von Keyserlingk MA 2020 The influence of different types of outdoor access on dairy cattle behavior. Frontiers in Veterinary Science 7: 257. 10.3389/fvets.2020.00257 32478110 PMC7238891

[r60] Ventura B, Weary DM and von Keyserlingk MA 2023 How might the public contribute to the discussion on cattle welfare? Perspectives of veterinarians and animal scientists. Animal Welfare 32: e69. 10.1017/awf.2023.88 38487462 PMC10936297

[r61] Wada S, Fukasawa M, Chiba T, Shishido T, Tozawa A and Ogura SI 2021 The effect of daily calf stroking frequency during the postnatal period on the establishment of the human-calf relationship. Animal Bioscience 34(10): 1717. 10.5713/ab.20.0745 33561326 PMC8495345

[r62] Waiblinger S, Boivin X, Pedersen V, Tosi MV, Janczak AM, Visser EK and Jones RB 2006 Assessing the human–animal relationship in farmed species: a critical review. Applied Animal Behaviour Science 101(3-4): 185–242. 10.1016/j.applanim.2006.02.001

[r63] Wellbrock W and Knierim A 2020 Evolution in animal husbandry–fitting animals, fitting systems, or fitting farmers? The role and agency of animal farmers in designing future animal production systems. Journal of Sustainable and Organic Agricultural Systems 70(1): 27–29. 10.3220/LBF1593104681000

[r64] Wibeck V, Dahlgren MA and Öberg G 2007 Learning in focus groups: An analytical dimension for enhancing focus group research. Qualitative Research 7(2): 249–267. 10.1177/1468794107076023

[r65] Wilkie R 2005 Sentient commodities and productive paradoxes: the ambiguous nature of human–livestock relations in Northeast Scotland. Journal of Rural Studies 21(2): 213–230. 10.1016/j.jrurstud.2004.10.002

[r66] Williams E, Sadler J, Rutter SM, Mancini C, Nawroth C, Neary JM, Ward SJ, Charlton G and Beaver A 2024 Human-animal interactions and machine-animal interactions in animals under human care: A summary of stakeholder and researcher perceptions and future directions. Animal Welfare 33: e27, 1–10. 10.1017/awf.2024.23 PMC11094549 38751800

[r67] Williamson K 2018 *Questionnaires, Individual Interviews and Focus Groups* . Research Methods, Second Edition, Information, Systems, and Contexts pp 379–403. Elsevier: London, UK. 10.1016/B978-0-08-102220-7.00016-9

